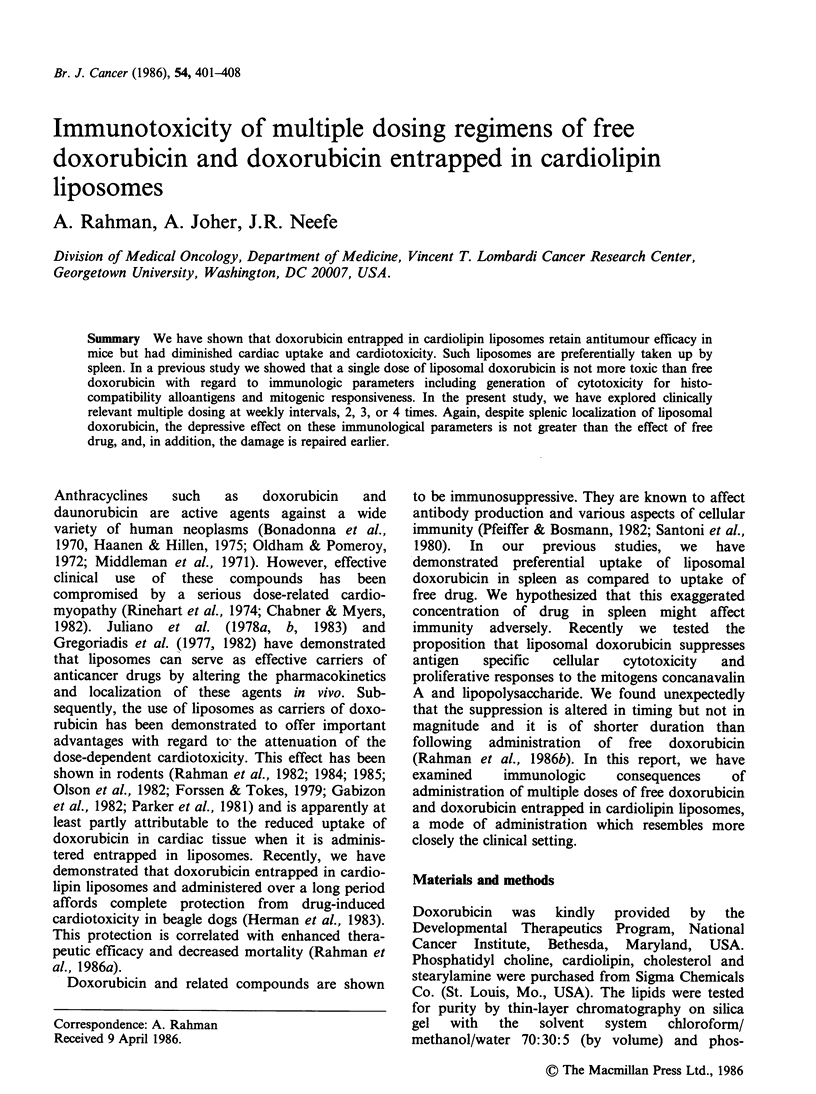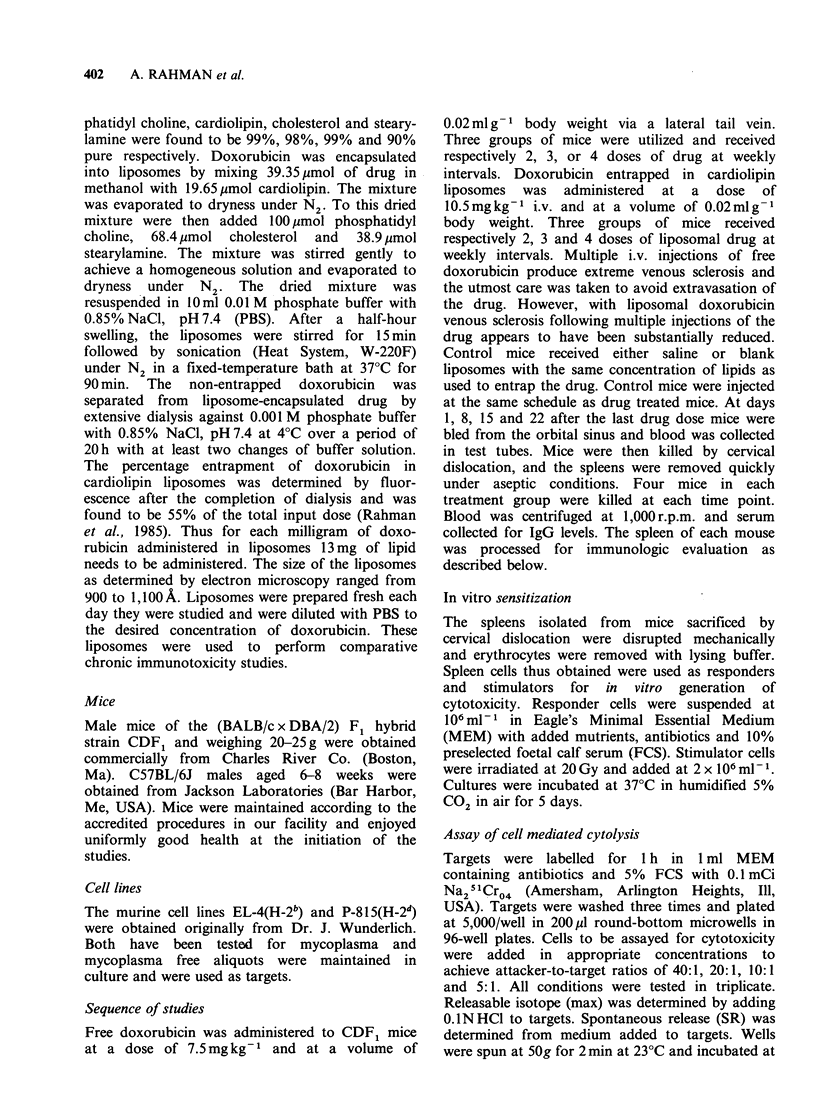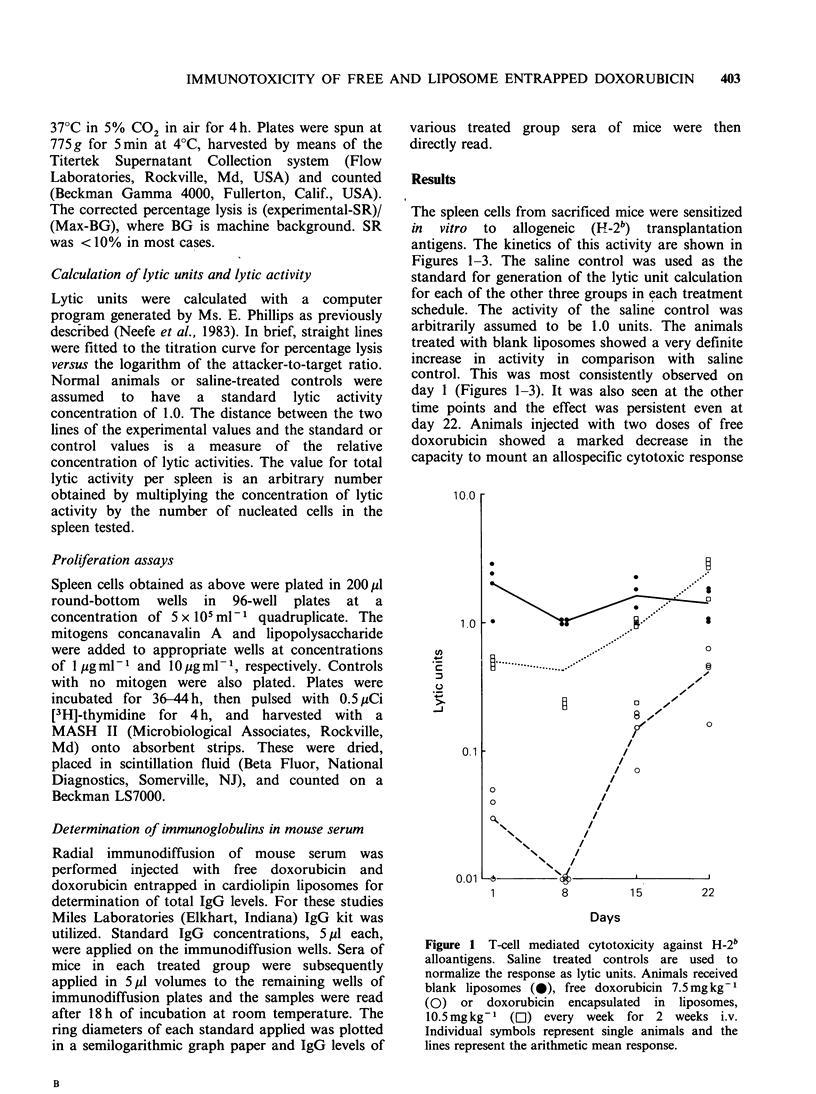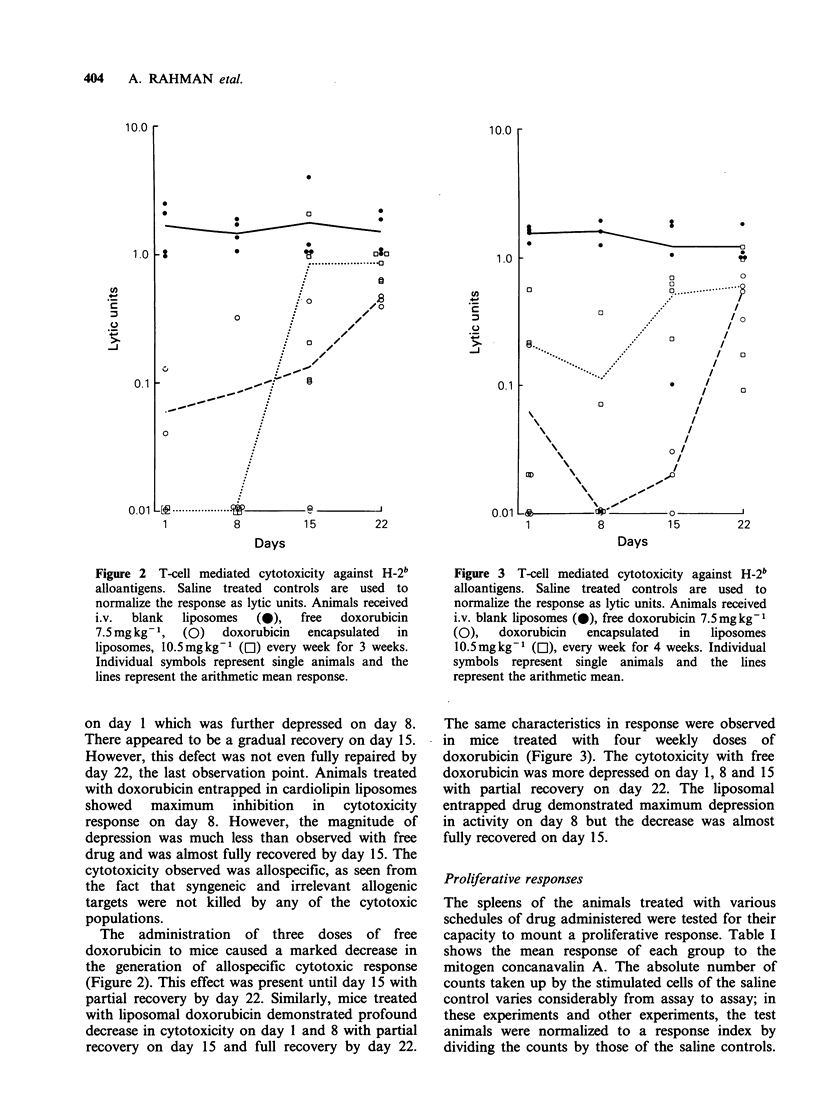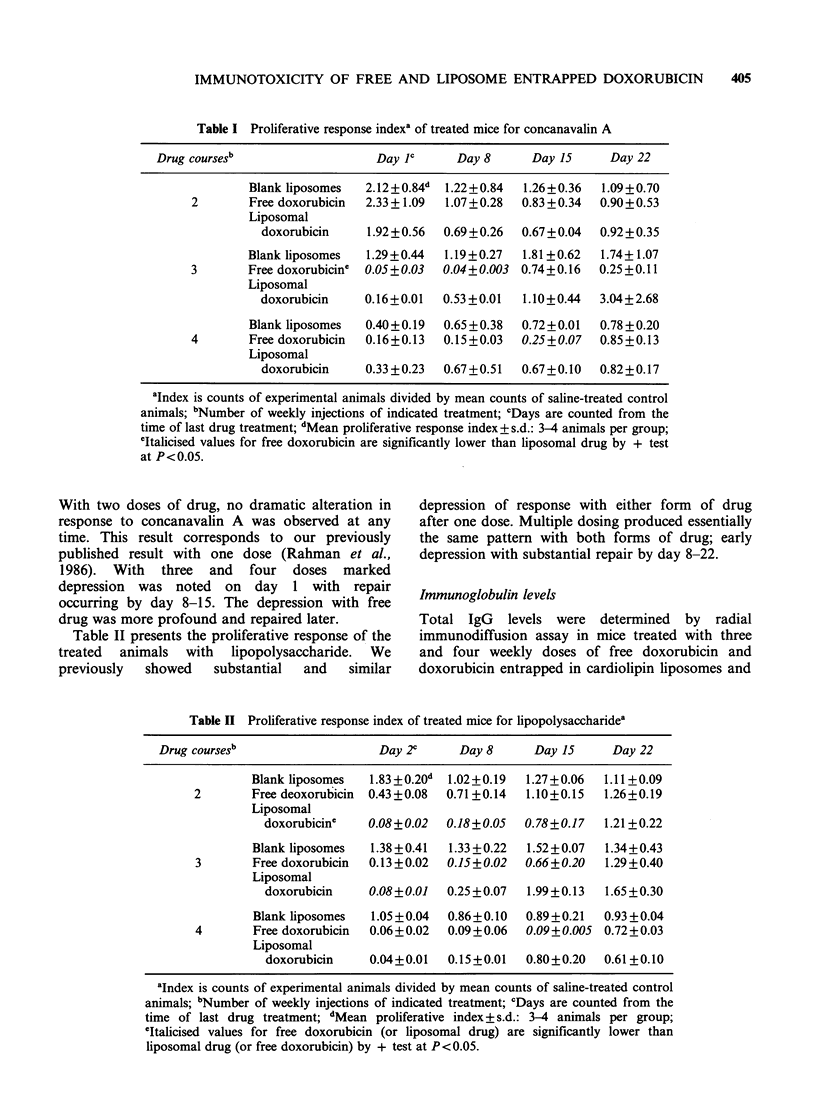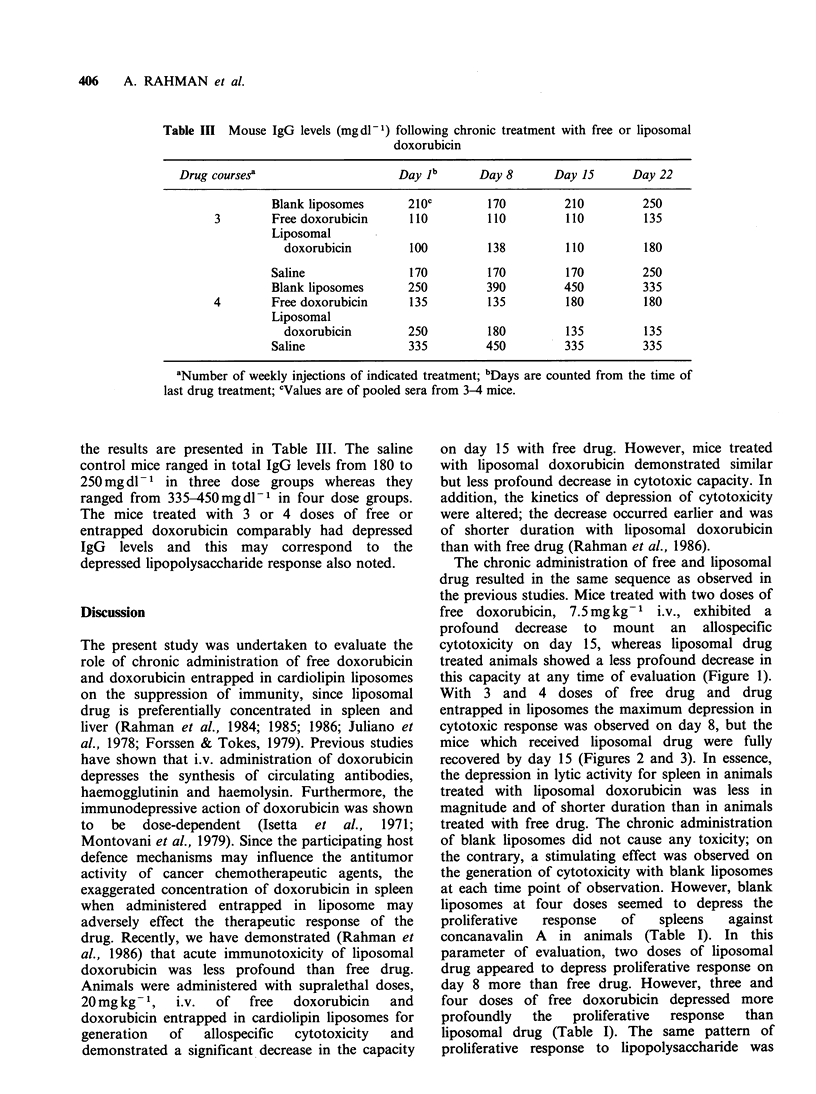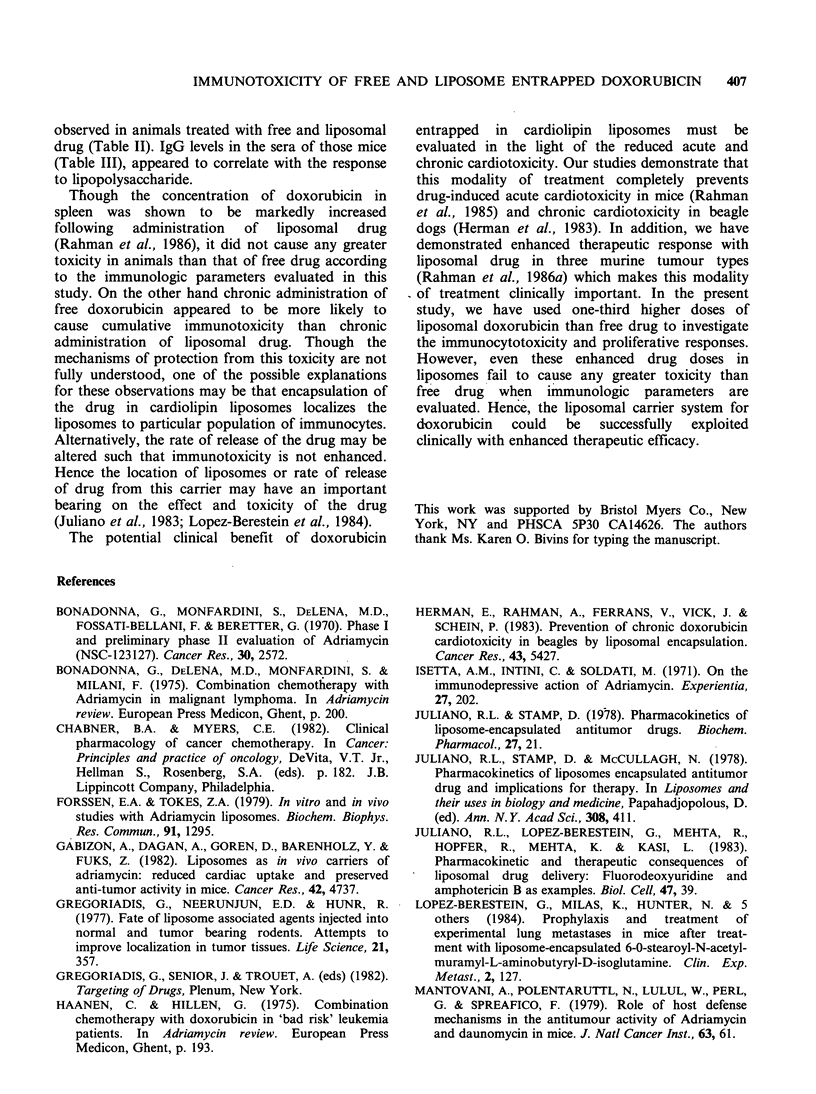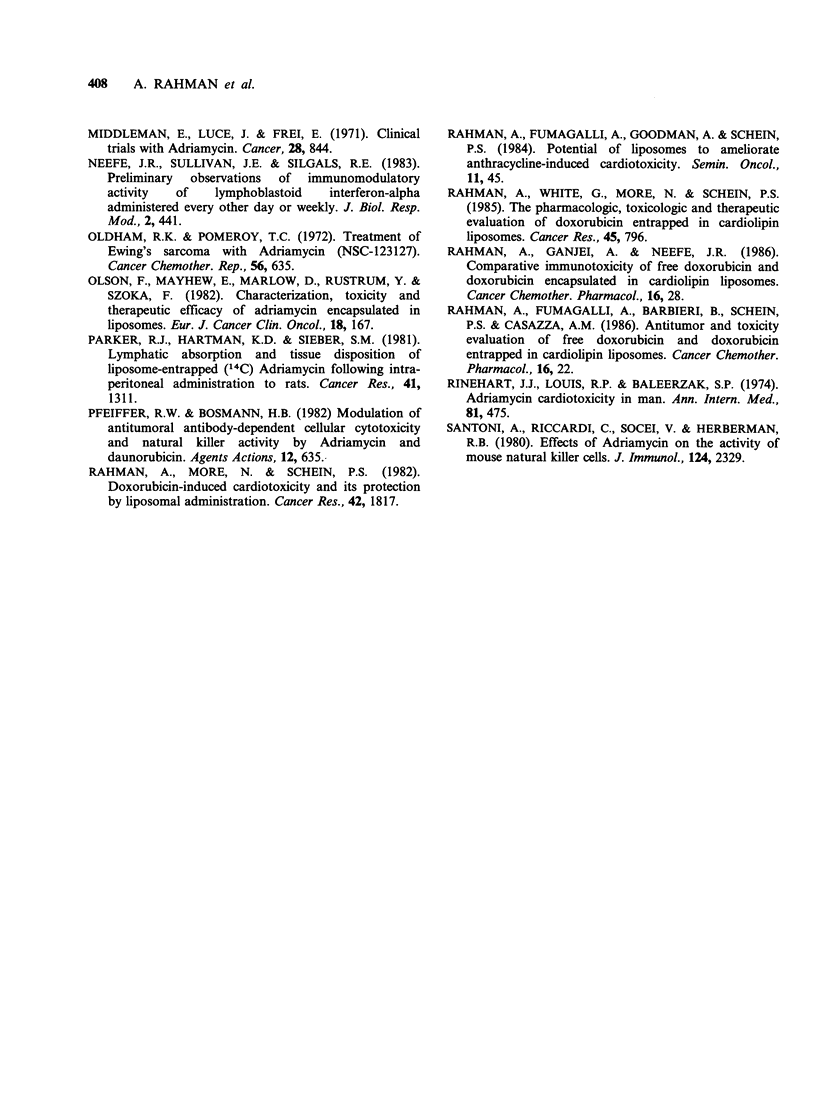# Immunotoxicity of multiple dosing regimens of free doxorubicin and doxorubicin entrapped in cardiolipin liposomes.

**DOI:** 10.1038/bjc.1986.190

**Published:** 1986-09

**Authors:** A. Rahman, A. Joher, J. R. Neefe

## Abstract

We have shown that doxorubicin entrapped in cardiolipin liposomes retain antitumour efficacy in mice but had diminished cardiac uptake and cardiotoxicity. Such liposomes are preferentially taken up by spleen. In a previous study we showed that a single dose of liposomal doxorubicin is not more toxic than free doxorubicin with regard to immunologic parameters including generation of cytotoxicity for histocompatibility alloantigens and mitogenic responsiveness. In the present study, we have explored clinically relevant multiple dosing at weekly intervals, 2, 3, or 4 times. Again, despite splenic localization of liposomal doxorubicin, the depressive effect on these immunological parameters is not greater than the effect of free drug, and, in addition, the damage is repaired earlier.


*Br. J. Cancer* (1986), **54**, 401–408

### Immunotoxicity of multiple dosing regimens of free doxorubicin and doxorubicin entrapped in cardiolipin liposomes

A. Rahman, A. Joher, J.R. Neefe

*Division of Medical Oncology, Department of Medicine, Vincent T. Lombardi Cancer Research Center, Georgetown University, Washington, DC 20007, USA.*

**Summary** We have shown that doxorubicin entrapped in cardiolipin liposomes retain antitumour efficacy in mice but had diminished cardiac uptake and cardiotoxicity. Such liposomes are preferentially taken up by spleen. In a previous study we showed that a single dose of liposomal doxorubicin is not more toxic than free doxorubicin with regard to immunologic parameters including generation of cytotoxicity for histocompatibility alloantigens and mitogenic responsiveness. In the present study, we have explored clinically relevant multiple dosing at weekly intervals, 2, 3, or 4 times. Again, despite splenic localization of liposomal doxorubicin, the depressive effect on these immunological parameters is not greater than the effect of free drug, and, in addition, the damage is repaired earlier.

Anthracyclines such as doxorubicin and daunorubicin are active agents against a wide variety of human neoplasms (Bonadonna *et al.*, 1970, Haanen & Hillen, 1975; Oldham & Pomeroy, 1972; Middleman *et al.*, 1971). However, effective clinical use of these compounds has been compromised by a serious dose-related cardiomyopathy (Rinehart *et al.*, 1974; Chabner & Myers, 1982). Juliano *et al.* (1978*a*, *b*, 1983) and Gregoriadis *et al.* (1977, 1982) have demonstrated that liposomes can serve as effective carriers of anticancer drugs by altering the pharmacokinetics and localization of these agents *in vivo*. Subsequently, the use of liposomes as carriers of doxorubicin has been demonstrated to offer important advantages with regard to the attenuation of the dose-dependent cardiotoxicity. This effect has been shown in rodents (Rahman *et al.*, 1982; 1984; 1985; Olson *et al.*, 1982; Forssen & Tokes, 1979; Gabizon *et al.*, 1982; Parker *et al.*, 1981) and is apparently at least partly attributable to the reduced uptake of doxorubicin in cardiac tissue when it is administered entrapped in liposomes. Recently, we have demonstrated that doxorubicin entrapped in cardiolipin liposomes and administered over a long period affords complete protection from drug-induced cardiotoxicity in beagle dogs (Herman *et al.*, 1983). This protection is correlated with enhanced therapeutic efficacy and decreased mortality (Rahman *et al.*, 1986*a*).

Doxorubicin and related compounds are shown to be immunosuppressive. They are known to affect antibody production and various aspects of cellular immunity (Pfeiffer & Bosmann, 1982; Santoni *et al.*, 1980). In our previous studies, we have demonstrated preferential uptake of liposomal doxorubicin in spleen as compared to uptake of free drug. We hypothesized that this exaggerated concentration of drug in spleen might affect immunity adversely. Recently we tested the proposition that liposomal doxorubicin suppresses antigen specific cellular cytotoxicity and proliferative responses to the mitogens concanavalin A and lipopolysaccharide. We found unexpectedly that the suppression is altered in timing but not in magnitude and it is of shorter duration than following administration of free doxorubicin (Rahman *et al.*, 1986*b*). In this report, we have examined immunologic consequences of administration of multiple doses of free doxorubicin and doxorubicin entrapped in cardiolipin liposomes, a mode of administration which resembles more closely the clinical setting.

### Materials and methods

Doxorubicin was kindly provided by the Developmental Therapeutics Program, National Cancer Institute, Bethesda, Maryland, USA. Phosphatidyl choline, cardiolipin, cholesterol and stearylamine were purchased from Sigma Chemicals Co. (St. Louis, Mo., USA). The lipids were tested for purity by thin-layer chromatography on silica gel with the solvent system chloroform/methanol/water 70:30:5 (by volume) and phos-

Correspondence: A. Rahman
Received 9 April 1986.

© The Macmillan Press Ltd., 1986

phatidyl choline, cardiolipin, cholesterol and stearylamine were found to be 99%, 98%, 99% and 90% pure respectively. Doxorubicin was encapsulated into liposomes by mixing 39.35 $\mu$mol of drug in methanol with 19.65 $\mu$mol cardiolipin. The mixture was evaporated to dryness under $N_2$. To this dried mixture were then added 100 $\mu$mol phosphatidyl choline, 68.4 $\mu$mol cholesterol and 38.9 $\mu$mol stearylamine. The mixture was stirred gently to achieve a homogeneous solution and evaporated to dryness under $N_2$. The dried mixture was resuspended in 10 ml 0.01 M phosphate buffer with 0.85% NaCl, pH 7.4 (PBS). After a half-hour swelling, the liposomes were stirred for 15 min followed by sonication (Heat System, W-220F) under $N_2$ in a fixed-temperature bath at 37°C for 90 min. The non-entrapped doxorubicin was separated from liposome-encapsulated drug by extensive dialysis against 0.001 M phosphate buffer with 0.85% NaCl, pH 7.4 at 4°C over a period of 20 h with at least two changes of buffer solution. The percentage entrapment of doxorubicin in cardiolipin liposomes was determined by fluorescence after the completion of dialysis and was found to be 55% of the total input dose (Rahman *et al.*, 1985). Thus for each milligram of doxorubicin administered in liposomes 13 mg of lipid needs to be administered. The size of the liposomes as determined by electron microscopy ranged from 900 to 1,100 Å. Liposomes were prepared fresh each day they were studied and were diluted with PBS to the desired concentration of doxorubicin. These liposomes were used to perform comparative chronic immunotoxicity studies.

### *Mice*

Male mice of the (BALB/c × DBA/2) $F_1$ hybrid strain $CDF_1$ and weighing 20–25 g were obtained commercially from Charles River Co. (Boston, Ma). C57BL/6J males aged 6–8 weeks were obtained from Jackson Laboratories (Bar Harbor, Me, USA). Mice were maintained according to the accredited procedures in our facility and enjoyed uniformly good health at the initiation of the studies.

### *Cell lines*

The murine cell lines EL-4(H-$2^b$) and P-815(H-$2^d$) were obtained originally from Dr. J. Wunderlich. Both have been tested for mycoplasma and mycoplasma free aliquots were maintained in culture and were used as targets.

### *Sequence of studies*

Free doxorubicin was administered to $CDF_1$ mice at a dose of 7.5 mg $kg^{-1}$ and at a volume of 0.02 ml $g^{-1}$ body weight via a lateral tail vein. Three groups of mice were utilized and received respectively 2, 3, or 4 doses of drug at weekly intervals. Doxorubicin entrapped in cardiolipin liposomes was administered at a dose of 10.5 mg $kg^{-1}$ i.v. and at a volume of 0.02 ml $g^{-1}$ body weight. Three groups of mice received respectively 2, 3 and 4 doses of liposomal drug at weekly intervals. Multiple i.v. injections of free doxorubicin produce extreme venous sclerosis and the utmost care was taken to avoid extravasation of the drug. However, with liposomal doxorubicin venous sclerosis following multiple injections of the drug appears to have been substantially reduced. Control mice received either saline or blank liposomes with the same concentration of lipids as used to entrap the drug. Control mice were injected at the same schedule as drug treated mice. At days 1, 8, 15 and 22 after the last drug dose mice were bled from the orbital sinus and blood was collected in test tubes. Mice were then killed by cervical dislocation, and the spleens were removed quickly under aseptic conditions. Four mice in each treatment group were killed at each time point. Blood was centrifuged at 1,000 r.p.m. and serum collected for IgG levels. The spleen of each mouse was processed for immunologic evaluation as described below.

### In vitro *sensitization*

The spleens isolated from mice sacrificed by cervical dislocation were disrupted mechanically and erythrocytes were removed with lysing buffer. Spleen cells thus obtained were used as responders and stimulators for *in vitro* generation of cytotoxicity. Responder cells were suspended at $10^6$ ml$^{-1}$ in Eagle's Minimal Essential Medium (MEM) with added mutrients, antibiotics and 10% preselected foetal calf serum (FCS). Stimulator cells were irradiated at 20 Gy and added at $2 \times 10^6$ ml$^{-1}$. Cultures were incubated at 37°C in humidified 5% $CO_2$ in air for 5 days.

### *Assay of cell mediated cytolysis*

Targets were labelled for 1 h in 1 ml MEM containing antibiotics and 5% FCS with 0.1 mCi $Na_2{}^{51}Cr_{04}$ (Amersham, Arlington Heights, Ill, USA). Targets were washed three times and plated at 5,000/well in 200 $\mu$l round-bottom microwells in 96-well plates. Cells to be assayed for cytotoxicity were added in appropriate concentrations to achieve attacker-to-target ratios of 40:1, 20:1, 10:1 and 5:1. All conditions were tested in triplicate. Releasable isotope (max) was determined by adding 0.1N HCl to targets. Spontaneous release (SR) was determined from medium added to targets. Wells were spun at 50*g* for 2 min at 23°C and incubated at

37°C in 5% $CO_2$ in air for 4 h. Plates were spun at 775 *g* for 5 min at 4°C, harvested by means of the Titertek Supernatant Collection system (Flow Laboratories, Rockville, Md, USA) and counted (Beckman Gamma 4000, Fullerton, Calif., USA). The corrected percentage lysis is (experimental-SR)/(Max-BG), where BG is machine background. SR was <10% in most cases.

*Calculation of lytic units and lytic activity*

Lytic units were calculated with a computer program generated by Ms. E. Phillips as previously described (Neefe *et al.*, 1983). In brief, straight lines were fitted to the titration curve for percentage lysis *versus* the logarithm of the attacker-to-target ratio. Normal animals or saline-treated controls were assumed to have a standard lytic activity concentration of 1.0. The distance between the two lines of the experimental values and the standard or control values is a measure of the relative concentration of lytic activities. The value for total lytic activity per spleen is an arbitrary number obtained by multiplying the concentration of lytic activity by the number of nucleated cells in the spleen tested.

*Proliferation assays*

Spleen cells obtained as above were plated in 200 $\mu$l round-bottom wells in 96-well plates at a concentration of $5 \times 10^5 ml^{-1}$ quadruplicate. The mitogens concanavalin A and lipopolysaccharide were added to appropriate wells at concentrations of 1 $\mu g ml^{-1}$ and 10 $\mu g ml^{-1}$, respectively. Controls with no mitogen were also plated. Plates were incubated for 36–44 h, then pulsed with 0.5 $\mu$Ci [$^3$H]-thymidine for 4 h, and harvested with a MASH II (Microbiological Associates, Rockville, Md) onto absorbent strips. These were dried, placed in scintillation fluid (Beta Fluor, National Diagnostics, Somerville, NJ), and counted on a Beckman LS7000.

*Determination of immunoglobulins in mouse serum*

Radial immunodiffusion of mouse serum was performed injected with free doxorubicin and doxorubicin entrapped in cardiolipin liposomes for determination of total IgG levels. For these studies Miles Laboratories (Elkhart, Indiana) IgG kit was utilized. Standard IgG concentrations, 5 $\mu$l each, were applied on the immunodiffusion wells. Sera of mice in each treated group were subsequently applied in 5 $\mu$l volumes to the remaining wells of immunodiffusion plates and the samples were read after 18 h of incubation at room temperature. The ring diameters of each standard applied was plotted in a semilogarithmic graph paper and IgG levels of various treated group sera of mice were then directly read.

### Results

The spleen cells from sacrificed mice were sensitized *in vitro* to allogeneic (H-2$^b$) transplantation antigens. The kinetics of this activity are shown in Figures 1–3. The saline control was used as the standard for generation of the lytic unit calculation for each of the other three groups in each treatment schedule. The activity of the saline control was arbitrarily assumed to be 1.0 units. The animals treated with blank liposomes showed a very definite increase in activity in comparison with saline control. This was most consistently observed on day 1 (Figures 1–3). It was also seen at the other time points and the effect was persistent even at day 22. Animals injected with two doses of free doxorubicin showed a marked decrease in the capacity to mount an allospecific cytotoxic response

**Figure 1** T-cell mediated cytotoxicity against H-2$^b$ alloantigens. Saline treated controls are used to normalize the response as lytic units. Animals received blank liposomes (●), free doxorubicin 7.5 mg $kg^{-1}$ (○) or doxorubicin encapsulated in liposomes, 10.5 mg $kg^{-1}$ (□) every week for 2 weeks i.v. Individual symbols represent single animals and the lines represent the arithmetic mean response.

**Figure 2** T-cell mediated cytotoxicity against H-$2^b$ alloantigens. Saline treated controls are used to normalize the response as lytic units. Animals received i.v. blank liposomes (●), free doxorubicin $7.5\,mg\,kg^{-1}$, (○) doxorubicin encapsulated in liposomes, $10.5\,mg\,kg^{-1}$ (□) every week for 3 weeks. Individual symbols represent single animals and the lines represent the arithmetic mean response.

**Figure 3** T-cell mediated cytotoxicity against H-$2^b$ alloantigens. Saline treated controls are used to normalize the response as lytic units. Animals received i.v. blank liposomes (●), free doxorubicin $7.5\,mg\,kg^{-1}$ (○), doxorubicin encapsulated in liposomes $10.5\,mg\,kg^{-1}$ (□), every week for 4 weeks. Individual symbols represent single animals and the lines represent the arithmetic mean.

on day 1 which was further depressed on day 8. There appeared to be a gradual recovery on day 15. However, this defect was not even fully repaired by day 22, the last observation point. Animals treated with doxorubicin entrapped in cardiolipin liposomes showed maximum inhibition in cytotoxicity response on day 8. However, the magnitude of depression was much less than observed with free drug and was almost fully recovered by day 15. The cytotoxicity observed was allospecific, as seen from the fact that syngeneic and irrelevant allogenic targets were not killed by any of the cytotoxic populations.

The administration of three doses of free doxorubicin to mice caused a marked decrease in the generation of allospecific cytotoxic response (Figure 2). This effect was present until day 15 with partial recovery by day 22. Similarly, mice treated with liposomal doxorubicin demonstrated profound decrease in cytotoxicity on day 1 and 8 with partial recovery on day 15 and full recovery by day 22.

The same characteristics in response were observed in mice treated with four weekly doses of doxorubicin (Figure 3). The cytotoxicity with free doxorubicin was more depressed on day 1, 8 and 15 with partial recovery on day 22. The liposomal entrapped drug demonstrated maximum depression in activity on day 8 but the decrease was almost fully recovered on day 15.

### *Proliferative responses*

The spleens of the animals treated with various schedules of drug administered were tested for their capacity to mount a proliferative response. Table I shows the mean response of each group to the mitogen concanavalin A. The absolute number of counts taken up by the stimulated cells of the saline control varies considerably from assay to assay; in these experiments and other experiments, the test animals were normalized to a response index by dividing the counts by those of the saline controls.

**Table I** Proliferative response index[a] of treated mice for concanavalin A

| *Drug courses*[b] | | *Day 1*[c] | *Day 8* | *Day 15* | *Day 22* |
|---|---|---|---|---|---|
| | Blank liposomes | 2.12±0.84[d] | 1.22±0.84 | 1.26±0.36 | 1.09±0.70 |
| 2 | Free doxorubicin | 2.33±1.09 | 1.07±0.28 | 0.83±0.34 | 0.90±0.53 |
| | Liposomal doxorubicin | 1.92±0.56 | 0.69±0.26 | 0.67±0.04 | 0.92±0.35 |
| | Blank liposomes | 1.29±0.44 | 1.19±0.27 | 1.81±0.62 | 1.74±1.07 |
| 3 | Free doxorubicin[e] | *0.05±0.03* | *0.04±0.003* | 0.74±0.16 | 0.25±0.11 |
| | Liposomal doxorubicin | 0.16±0.01 | 0.53±0.01 | 1.10±0.44 | 3.04±2.68 |
| | Blank liposomes | 0.40±0.19 | 0.65±0.38 | 0.72±0.01 | 0.78±0.20 |
| 4 | Free doxorubicin | 0.16±0.13 | 0.15±0.03 | *0.25±0.07* | 0.85±0.13 |
| | Liposomal doxorubicin | 0.33±0.23 | 0.67±0.51 | 0.67±0.10 | 0.82±0.17 |

[a]Index is counts of experimental animals divided by mean counts of saline-treated control animals; [b]Number of weekly injections of indicated treatment; [c]Days are counted from the time of last drug treatment; [d]Mean proliferative response index±s.d.: 3–4 animals per group; [e]Italicised values for free doxorubicin are significantly lower than liposomal drug by + test at $P<0.05$.

With two doses of drug, no dramatic alteration in response to concanavalin A was observed at any time. This result corresponds to our previously published result with one dose (Rahman *et al.*, 1986). With three and four doses marked depression was noted on day 1 with repair occurring by day 8–15. The depression with free drug was more profound and repaired later.

Table II presents the proliferative response of the treated animals with lipopolysaccharide. We previously showed substantial and similar depression of response with either form of drug after one dose. Multiple dosing produced essentially the same pattern with both forms of drug; early depression with substantial repair by day 8–22.

### *Immunoglobulin levels*

Total IgG levels were determined by radial immunodiffusion assay in mice treated with three and four weekly doses of free doxorubicin and doxorubicin entrapped in cardiolipin liposomes and

**Table II** Proliferative response index of treated mice for lipopolysaccharide[a]

| *Drug courses*[b] | | *Day 2*[c] | *Day 8* | *Day 15* | *Day 22* |
|---|---|---|---|---|---|
| | Blank liposomes | 1.83±0.20[d] | 1.02±0.19 | 1.27±0.06 | 1.11±0.09 |
| 2 | Free deoxorubicin | 0.43±0.08 | 0.71±0.14 | 1.10±0.15 | 1.26±0.19 |
| | Liposomal doxorubicin[e] | *0.08±0.02* | *0.18±0.05* | *0.78±0.17* | 1.21±0.22 |
| | Blank liposomes | 1.38±0.41 | 1.33±0.22 | 1.52±0.07 | 1.34±0.43 |
| 3 | Free doxorubicin | 0.13±0.02 | *0.15±0.02* | *0.66±0.20* | 1.29±0.40 |
| | Liposomal doxorubicin | *0.08±0.01* | 0.25±0.07 | 1.99±0.13 | 1.65±0.30 |
| | Blank liposomes | 1.05±0.04 | 0.86±0.10 | 0.89±0.21 | 0.93±0.04 |
| 4 | Free doxorubicin | 0.06±0.02 | 0.09±0.06 | *0.09±0.005* | 0.72±0.03 |
| | Liposomal doxorubicin | 0.04±0.01 | 0.15±0.01 | 0.80±0.20 | 0.61±0.10 |

[a]Index is counts of experimental animals divided by mean counts of saline-treated control animals; [b]Number of weekly injections of indicated treatment; [c]Days are counted from the time of last drug treatment; [d]Mean proliferative index±s.d.: 3–4 animals per group; [e]Italicised values for free doxorubicin (or liposomal drug) are significantly lower than liposomal drug (or free doxorubicin) by + test at $P<0.05$.

**Table III** Mouse IgG levels ($mg\,dl^{-1}$) following chronic treatment with free or liposomal doxorubicin

| *Drug courses*[a] | | *Day 1*[b] | *Day 8* | *Day 15* | *Day 22* |
|---|---|---|---|---|---|
| | Blank liposomes | 210[c] | 170 | 210 | 250 |
| 3 | Free doxorubicin | 110 | 110 | 110 | 135 |
| | Liposomal doxorubicin | 100 | 138 | 110 | 180 |
| | Saline | 170 | 170 | 170 | 250 |
| | Blank liposomes | 250 | 390 | 450 | 335 |
| 4 | Free doxorubicin | 135 | 135 | 180 | 180 |
| | Liposomal doxorubicin | 250 | 180 | 135 | 135 |
| | Saline | 335 | 450 | 335 | 335 |

[a]Number of weekly injections of indicated treatment; [b]Days are counted from the time of last drug treatment; [c]Values are of pooled sera from 3–4 mice.

the results are presented in Table III. The saline control mice ranged in total IgG levels from 180 to $250\,mg\,dl^{-1}$ in three dose groups whereas they ranged from $335–450\,mg\,dl^{-1}$ in four dose groups. The mice treated with 3 or 4 doses of free or entrapped doxorubicin comparably had depressed IgG levels and this may correspond to the depressed lipopolysaccharide response also noted.

### Discussion

The present study was undertaken to evaluate the role of chronic administration of free doxorubicin and doxorubicin entrapped in cardiolipin liposomes on the suppression of immunity, since liposomal drug is preferentially concentrated in spleen and liver (Rahman *et al.*, 1984; 1985; 1986; Juliano *et al.*, 1978; Forssen & Tokes, 1979). Previous studies have shown that i.v. administration of doxorubicin depresses the synthesis of circulating antibodies, haemogglutinin and haemolysin. Furthermore, the immunodepressive action of doxorubicin was shown to be dose-dependent (Isetta *et al.*, 1971; Montovani *et al.*, 1979). Since the participating host defence mechanisms may influence the antitumor activity of cancer chemotherapeutic agents, the exaggerated concentration of doxorubicin in spleen when administered entrapped in liposome may adversely effect the therapeutic response of the drug. Recently, we have demonstrated (Rahman *et al.*, 1986) that acute immunotoxicity of liposomal doxorubicin was less profound than free drug. Animals were administered with supralethal doses, $20\,mg\,kg^{-1}$, i.v. of free doxorubicin and doxorubicin entrapped in cardiolipin liposomes for generation of allospecific cytotoxicity and demonstrated a significant decrease in the capacity on day 15 with free drug. However, mice treated with liposomal doxorubicin demonstrated similar but less profound decrease in cytotoxic capacity. In addition, the kinetics of depression of cytotoxicity were altered; the decrease occurred earlier and was of shorter duration with liposomal doxorubicin than with free drug (Rahman *et al.*, 1986).

The chronic administration of free and liposomal drug resulted in the same sequence as observed in the previous studies. Mice treated with two doses of free doxorubicin, $7.5\,mg\,kg^{-1}$ i.v., exhibited a profound decrease to mount an allospecific cytotoxicity on day 15, whereas liposomal drug treated animals showed a less profound decrease in this capacity at any time of evaluation (Figure 1). With 3 and 4 doses of free drug and drug entrapped in liposomes the maximum depression in cytotoxic response was observed on day 8, but the mice which received liposomal drug were fully recovered by day 15 (Figures 2 and 3). In essence, the depression in lytic activity for spleen in animals treated with liposomal doxorubicin was less in magnitude and of shorter duration than in animals treated with free drug. The chronic administration of blank liposomes did not cause any toxicity; on the contrary, a stimulating effect was observed on the generation of cytotoxicity with blank liposomes at each time point of observation. However, blank liposomes at four doses seemed to depress the proliferative response of spleens against concanavalin A in animals (Table I). In this parameter of evaluation, two doses of liposomal drug appeared to depress proliferative response on day 8 more than free drug. However, three and four doses of free doxorubicin depressed more profoundly the proliferative response than liposomal drug (Table I). The same pattern of proliferative response to lipopolysaccharide was observed in animals treated with free and liposomal drug (Table II). IgG levels in the sera of those mice (Table III), appeared to correlate with the response to lipopolysaccharide.

Though the concentration of doxorubicin in spleen was shown to be markedly increased following administration of liposomal drug (Rahman *et al.*, 1986), it did not cause any greater toxicity in animals than that of free drug according to the immunologic parameters evaluated in this study. On the other hand chronic administration of free doxorubicin appeared to be more likely to cause cumulative immunotoxicity than chronic administration of liposomal drug. Though the mechanisms of protection from this toxicity are not fully understood, one of the possible explanations for these observations may be that encapsulation of the drug in cardiolipin liposomes localizes the liposomes to particular population of immunocytes. Alternatively, the rate of release of the drug may be altered such that immunotoxicity is not enhanced. Hence the location of liposomes or rate of release of drug from this carrier may have an important bearing on the effect and toxicity of the drug (Juliano *et al.*, 1983; Lopez-Berestein *et al.*, 1984).

The potential clinical benefit of doxorubicin entrapped in cardiolipin liposomes must be evaluated in the light of the reduced acute and chronic cardiotoxicity. Our studies demonstrate that this modality of treatment completely prevents drug-induced acute cardiotoxicity in mice (Rahman *et al.*, 1985) and chronic cardiotoxicity in beagle dogs (Herman *et al.*, 1983). In addition, we have demonstrated enhanced therapeutic response with liposomal drug in three murine tumour types (Rahman *et al.*, 1986*a*) which makes this modality of treatment clinically important. In the present study, we have used one-third higher doses of liposomal doxorubicin than free drug to investigate the immunocytotoxicity and proliferative responses. However, even these enhanced drug doses in liposomes fail to cause any greater toxicity than free drug when immunologic parameters are evaluated. Hence, the liposomal carrier system for doxorubicin could be successfully exploited clinically with enhanced therapeutic efficacy.

This work was supported by Bristol Myers Co., New York, NY and PHSCA 5P30 CA14626. The authors thank Ms. Karen O. Bivins for typing the manuscript.

### References

BONADONNA, G., MONFARDINI, S., DELENA, M.D., FOSSATI-BELLANI, F. & BERETTER, G. (1970). Phase I and preliminary phase II evaluation of Adriamycin (NSC-123127). *Cancer Res.*, **30**, 2572.

BONADONNA, G., DELENA, M.D., MONFARDINI, S. & MILANI, F. (1975). Combination chemotherapy with Adriamycin in malignant lymphoma. In *Adriamycin review*. European Press Medicon, Ghent, p. 200.

CHABNER, B.A. & MYERS, C.E. (1982). Clinical pharmacology of cancer chemotherapy. In *Cancer: Principles and practice of oncology*, DeVita, V.T. Jr., Hellman S., Rosenberg, S.A. (eds). p. 182. J.B. Lippincott Company, Philadelphia.

FORSSEN, E.A. & TOKES, Z.A. (1979). *In vitro* and *in vivo* studies with Adriamycin liposomes. *Biochem. Biophys. Res. Commun.*, **91**, 1295.

GABIZON, A., DAGAN, A., GOREN, D., BARENHOLZ, Y. & FUKS, Z. (1982). Liposomes as *in vivo* carriers of adriamycin: reduced cardiac uptake and preserved anti-tumor activity in mice. *Cancer Res.*, **42**, 4737.

GREGORIADIS, G., NEERUNJUN, E.D. & HUNR, R. (1977). Fate of liposome associated agents injected into normal and tumor bearing rodents. Attempts to improve localization in tumor tissues. *Life Science*, **21**, 357.

GREGORIADIS, G., SENIOR, J. & TROUET, A. (eds) (1982). *Targeting of Drugs*, Plenum, New York.

HAANEN, C. & HILLEN, G. (1975). Combination chemotherapy with doxorubicin in 'bad risk' leukemia patients. In *Adriamycin review*. European Press Medicon, Ghent, p. 193.

HERMAN, E., RAHMAN, A., FERRANS, V., VICK, J. & SCHEIN, P. (1983). Prevention of chronic doxorubicin cardiotoxicity in beagles by liposomal encapsulation. *Cancer Res.*, **43**, 5427.

ISETTA, A.M., INTINI, C. & SOLDATI, M. (1971). On the immunodepressive action of Adriamycin. *Experientia*, **27**, 202.

JULIANO, R.L. & STAMP, D. (1978). Pharmacokinetics of liposome-encapsulated antitumor drugs. *Biochem. Pharmacol.*, **27**, 21.

JULIANO, R.L., STAMP, D. & MCCULLAGH, N. (1978). Pharmacokinetics of liposomes encapsulated antitumor drug and implications for therapy. In *Liposomes and their uses in biology and medicine*, Papahadjopolous, D. (ed). *Ann. N.Y. Acad Sci.*, **308**, 411.

JULIANO, R.L., LOPEZ-BERESTEIN, G., MEHTA, R., HOPFER, R., MEHTA, K. & KASI, L. (1983). Pharmacokinetic and therapeutic consequences of liposomal drug delivery: Fluorodeoxyuridine and amphotericin B as examples. *Biol. Cell*, **47**, 39.

LOPEZ-BERESTEIN, G., MILAS, K., HUNTER, N. & 5 others (1984). Prophylaxis and treatment of experimental lung metastases in mice after treatment with liposome-encapsulated 6-0-stearoyl-N-acetyl-muramyl-L-aminobutyryl-D-isoglutamine. *Clin. Exp. Metast.*, **2**, 127.

MANTOVANI, A., POLENTARUTTL, N., LULUL, W., PERL, G. & SPREAFICO, F. (1979). Role of host defense mechanisms in the antitumour activity of Adriamycin and daunomycin in mice. *J. Natl Cancer Inst.*, **63**, 61.

MIDDLEMAN, E., LUCE, J. & FREI, E. (1971). Clinical trials with Adriamycin. *Cancer*, **28**, 844.

NEEFE, J.R., SULLIVAN, J.E. & SILGALS, R.E. (1983). Preliminary observations of immunomodulatory activity of lymphoblastoid interferon-alpha administered every other day or weekly. *J. Biol. Resp. Mod.*, **2**, 441.

OLDHAM, R.K. & POMEROY, T.C. (1972). Treatment of Ewing's sarcoma with Adriamycin (NSC-123127). *Cancer Chemother. Rep.*, **56**, 635.

OLSON, F., MAYHEW, E., MARLOW, D., RUSTRUM, Y. & SZOKA, F. (1982). Characterization, toxicity and therapeutic efficacy of adriamycin encapsulated in liposomes. *Eur. J. Cancer Clin. Oncol.*, **18**, 167.

PARKER, R.J., HARTMAN, K.D. & SIEBER, S.M. (1981). Lymphatic absorption and tissue disposition of liposome-entrapped ($^{14}$C) Adriamycin following intraperitoneal administration to rats. *Cancer Res.*, **41**, 1311.

PFEIFFER, R.W. & BOSMANN, H.B. (1982) Modulation of antitumoral antibody-dependent cellular cytotoxicity and natural killer activity by Adriamycin and daunorubicin. *Agents Actions*, **12**, 635.

RAHMAN, A., MORE, N. & SCHEIN, P.S. (1982). Doxorubicin-induced cardiotoxicity and its protection by liposomal administration. *Cancer Res.*, **42**, 1817.

RAHMAN, A., FUMAGALLI, A., GOODMAN, A. & SCHEIN, P.S. (1984). Potential of liposomes to ameliorate anthracycline-induced cardiotoxicity. *Semin. Oncol.*, **11**, 45.

RAHMAN, A., WHITE, G., MORE, N. & SCHEIN, P.S. (1985). The pharmacologic, toxicologic and therapeutic evaluation of doxorubicin entrapped in cardiolipin liposomes. *Cancer Res.*, **45**, 796.

RAHMAN, A., GANJEI, A. & NEEFE, J.R. (1986). Comparative immunotoxicity of free doxorubicin and doxorubicin encapsulated in cardiolipin liposomes. *Cancer Chemother. Pharmacol.*, **16**, 28.

RAHMAN, A., FUMAGALLI, A., BARBIERI, B., SCHEIN, P.S. & CASAZZA, A.M. (1986). Antitumor and toxicity evaluation of free doxorubicin and doxorubicin entrapped in cardiolipin liposomes. *Cancer Chemother. Pharmacol.*, **16**, 22.

RINEHART, J.J., LOUIS, R.P. & BALEERZAK, S.P. (1974). Adriamycin cardiotoxicity in man. *Ann. Intern. Med.*, **81**, 475.

SANTONI, A., RICCARDI, C., SOCEI, V. & HERBERMAN, R.B. (1980). Effects of Adriamycin on the activity of mouse natural killer cells. *J. Immunol.*, **124**, 2329.